# Barriers and facilitators to treat-to-target in axial spondyloarthritis in clinical practice: a mixed methods study

**DOI:** 10.1007/s00296-025-05795-6

**Published:** 2025-01-31

**Authors:** Marius L. Smits, Casper Webers, Mirte van Dooren, Elien A. M. Mahler, Johanna E. Vriezekolk, Astrid van Tubergen

**Affiliations:** 1 Department of Rheumatology, Maastricht University Medical Centre+, P.O. Box 5800, Maastricht, 6202 AZ The Netherlands; 2 Care and Public Health Research Institute (CAPHRI), Maastricht University, Maastricht, The Netherlands; 3 Department of Rheumatology, Sint Maartenskliniek, Nijmegen, The Netherlands

**Keywords:** Axial spondyloarthritis, Attitude of health personnel, Attitude to health, Delivery of health care, Quality of health care

## Abstract

This study aimed to explore the perceptions of patients and rheumatologists about a treat-to-target (T2T) strategy in axial spondyloarthritis (axSpA) and identify the barriers and facilitators to its implementation in clinical practice. A mixed methods design was applied. Patients with axSpA who visited the outpatient clinic with active disease (AxSpA Disease Activity Score [ASDAS] ≥ 2.1), but did not receive a treatment adjustment, were identified. These patient cases were discussed in individual semi-structured interviews with the respective treating rheumatologists, and a subgroup of these patients was also interviewed. In parallel, all interviewed participants completed a quantitative survey. Qualitative and quantitative data were analysed thematically and descriptively, respectively. Twenty-three patients were discussed with 11 rheumatologists, and 16 of these patients were interviewed personally. Barriers to T2T included challenges in the measurement of inflammatory disease activity using the ASDAS, and numerous patient-related factors such as concern about treatment adaptations. The limited number of viable treatment options and scarce amount of evidence supporting T2T in axSpA, as well as logistical challenges, were additional obstacles. Facilitators included patients’ broad knowledge about axSpA, rheumatologists’ awareness of T2T recommendations, and positive doctor-patient relationships with the application of shared decision-making. Moreover, a supporting infrastructure, such as one with high accessibility to the outpatient clinic between scheduled visits, was considered necessary for the application of a T2T strategy. In conclusion, numerous barriers and facilitators to the implementation of a T2T strategy in axSpA are present, which need to be considered when applying this treatment approach in clinical practice.

**Supplementary Information:**

The online version contains supplementary material available at 10.1007/s00296-025-05795-6.

## Introduction

In axial spondyloarthritis (axSpA), the adequate management of disease activity is important to maintain the physical and psychosocial functioning of patients, and to prevent complications [1]. Previous studies have demonstrated an association between persistent disease and the progression of irreversible structural damage, increased use of healthcare resources, and higher societal costs due to reduced work productivity [2–4].

In view of these considerations, a treatment approach that has gained increasing attention is the ‘treat-to-target’ (T2T) strategy [5–7]. This approach firstly entails determining a treatment target, typically inactive disease (ID) or low disease activity (LDA), and the swift initiation of pharmacological therapy as necessary. Subsequently, the target is regularly evaluated (‘tight control’), and treatment is adapted until the target is reached [8]. Throughout, the rheumatologist employs shared decision-making (SDM) with the patient to ensure that treatment targets align with the patient’s preferences [8]. Successful implementation of a T2T strategy therefore requires synergy between patients, rheumatologists, and the organisation of care [9].

In rheumatoid arthritis (RA) and psoriatic arthritis, T2T strategies have been effective in improving patient outcomes [6, 7, 10]. In axSpA, however, evidence remains inconclusive. Most notably, the Tight Control in Spondyloarthritis (TICOSPA) trial, which compared a T2T strategy aiming for LDA with usual care in patients with axSpA, did not reach its primary endpoint [11]. Nonetheless, numerous secondary endpoints showed a general trend in favour of this T2T strategy [11]. As such, several international recommendations endorse the application of T2T strategies in axSpA [8, 12].

Even with the increased recognition of the potential value of a T2T strategy in axSpA, application in clinical practice is suboptimal. Illustratively, a Dutch observational study reported that while disease activity data were available for 84% of the axSpA patient population, only 38% had achieved ID/LDA [13]. Furthermore, another study highlighted that agreement with T2T recommendations amongst rheumatologists in the context of RA did not necessarily translate into implementation in practice [14]. Currently, knowledge about factors driving this discordance is lacking [15, 16]. To facilitate successful implementation, a better understanding of elements that limit the application of a T2T strategy (barriers), and enabling factors that are already present in clinical practice (facilitators), is necessary. This study therefore aimed to explore the perceptions of patients and rheumatologists about a T2T strategy in axSpA, and to identify the barriers and facilitators associated with its implementation.

## Methods

### Study design

This study applied a convergent parallel mixed methods design [17]. Both patients with axSpA and their treating rheumatologists from two Dutch hospitals (Maastricht University Medical Centre+ and Sint Maartenskliniek) were invited for an individual, semi-structured interview, and to complete a supplementary quantitative survey immediately afterwards.

### Participants

Inclusion took place between October 2023 and April 2024. All patients of the participating centres who visited the rheumatology outpatient clinic were screened against the following inclusion criteria: (1) clinical diagnosis of axSpA, (2) an ASDAS indicating active disease (≥ 2.1) calculated during an in-person consultation, and (3) no treatment adaptations made by the treating rheumatologist during that consultation, despite an ASDAS ≥ 2.1. Treatment adaptation was defined as a change to the dose or frequency of existing anti-inflammatory drugs, or a switch to, or addition of, a new anti-inflammatory drug.

Interviews were planned with both the treating rheumatologists of the identified patients and a subgroup of these patients (those who were willing to participate) at the earliest possible opportunity after the in-person consultation.

### Qualitative data

Separate interview guides for patients and rheumatologists were developed (Online Resource 1 and 2). Interviews with patients focused on their individual disease characteristics, current treatment, knowledge of the T2T strategy, and views on SDM. Rheumatologists were interviewed on their (shared) decision-making process regarding treatment adaptations, centred specifically on their patients who had also been interviewed. To ensure that a sufficient number and variety of patients were covered, several additional (non-participating) patients that the included rheumatologists had recently seen at the outpatient clinic who met the inclusion criteria were also discussed. Prior to the interviews, rheumatologists were informed about the patients to be discussed to allow for adequate preparation.

All participants were interviewed by one researcher (MS) not involved in the patients’ care. Interviews were audio recorded, transcribed verbatim and anonymised. Analysis started with the reading and re-reading of all transcripts to gain familiarity with the contents. Thematic analysis was then performed by the same researcher who conducted the interviews according to grounded theory using NVivo V.14 (QSR International Pty Ltd, Burlington, MA, USA), in ongoing collaboration with the research team [18]. This entailed the inductive coding of key concepts, followed by the organisation of the codes into themes from which conclusions were formed. Finally, representative quotes were selected to illustrate the findings, with reference to the Consolidated Criteria for Reporting Qualitative Research (COREQ) checklist [19].

### Quantitative data

Separate surveys for patients and rheumatologists were composed (Online Resource 3 and 4). The surveys focused on the usefulness of the available tools for disease activity measurement and the current treatment options for axSpA. Additionally, rheumatologists evaluated the concept of T2T.

Responses were analysed descriptively using Microsoft Excel (Microsoft Corp., Redmond, VA, USA). Lastly, the quantitative and qualitative results were integrated by a weaving approach after discussions with the research team [17].

### Ethics

The Medical Research Ethics Committee of the Academic Hospital Maastricht/Maastricht University determined that the Dutch Medical Research Involving Human Subjects Act was non-applicable to this study (METC azM/UM 2023 − 0213). All participants provided informed consent for their provided data to be used for research purposes.

## Results

Forty patients were invited, of which 16 participated. Reasons for non-participation included time constraints, low motivation, and the presence of restricting comorbidities. Participating patients had a mean age of 57.4 (SD 13.3) years, mean symptom duration of 26.8 (SD 17.4, range 3–64) years, and mean ASDAS of 3.0 (SD 0.5) (Table 1). The 11 included rheumatologists had a mean work experience of 16.7 (SD 10.6) years, and 7 (63.6%) worked in a university hospital. A total of 23 patient cases were discussed with the rheumatologists (1–4 patients each): the 16 interviewed patients and seven additional non-participating patients. The characteristics of patients in both groups were largely comparable. Fifteen of the 23 (65.2%) patients discussed were being treated with a biological disease-modifying anti-rheumatic drug (bDMARD) at the time of the consultation with no treatment adjustments made.

**Table 1:** Characteristics of the participating patients and rheumatologists

Characteristics	Patients (*n* = 16)	Rheumatologists (*n* = 11)
Age, years	57.4 (13.3)	49.6 (8.2)
Female sex, n (%)	8 (50.0)	9 (81.8)
Type of healthcare institution, n (%)		
University hospital	10 (62.5)	7 (63.6)
Specialised MSK hospital	6 (37.5)	4 (36.4)
Symptom duration, years	26.8 (17.4)	NA
Current bDMARD use, n (%)	11 (68.8)	NA
ASDAS at inclusion	3.0 (0.5)	NA
Work experience, years	NA	16.7 (10.6)

Values are presented as mean (standard deviation), unless otherwise indicated

ASDAS: Axial Spondyloarthritis Disease Activity Score; bDMARD: biological disease-modifying anti-rheumatic drug; MSK: musculoskeletal; NA: not applicable

**Fig. 1:**
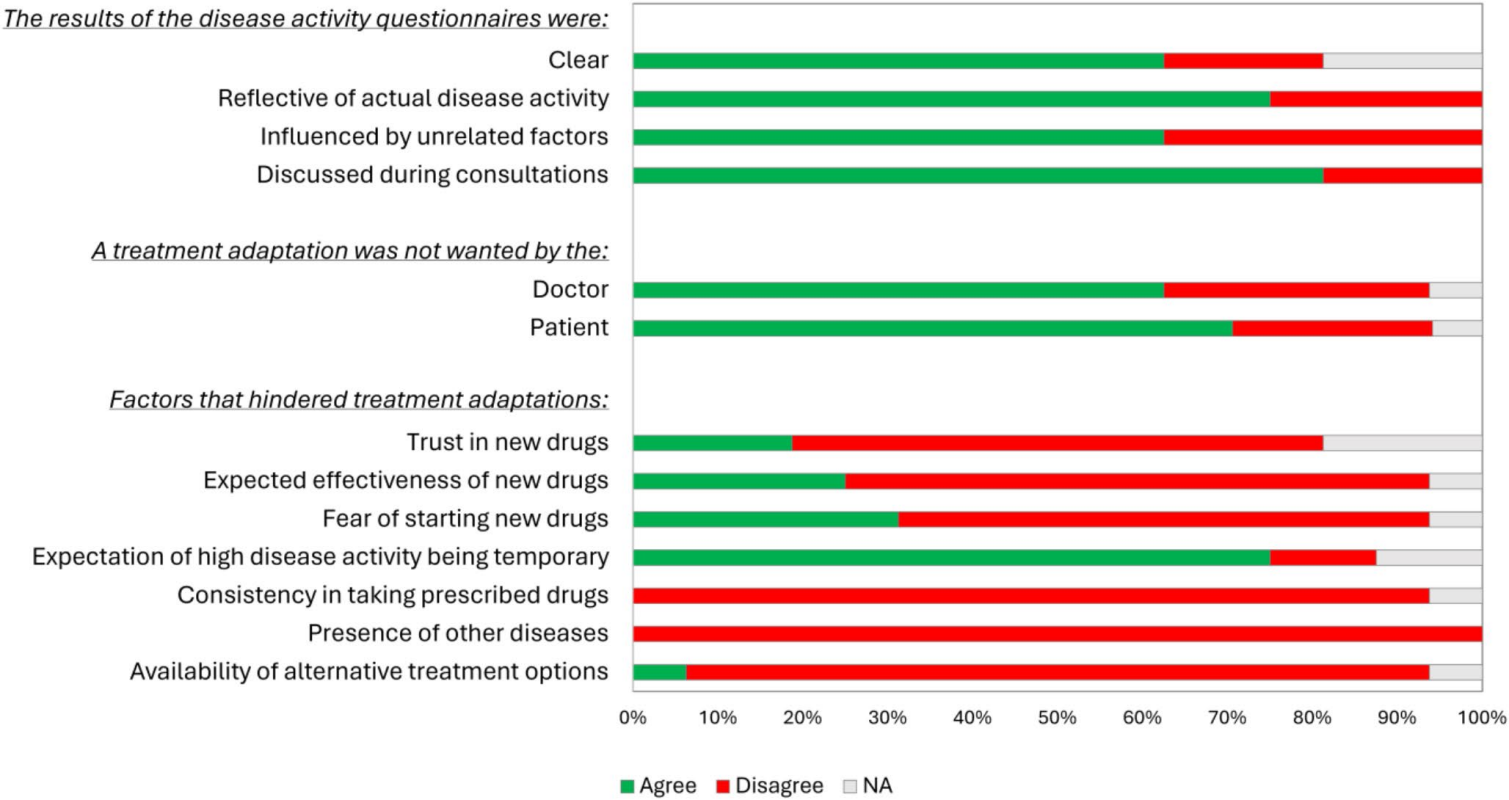
Results of the supplementary survey for patients (*n* = 16). NA: not applicable

**Fig. 2:**
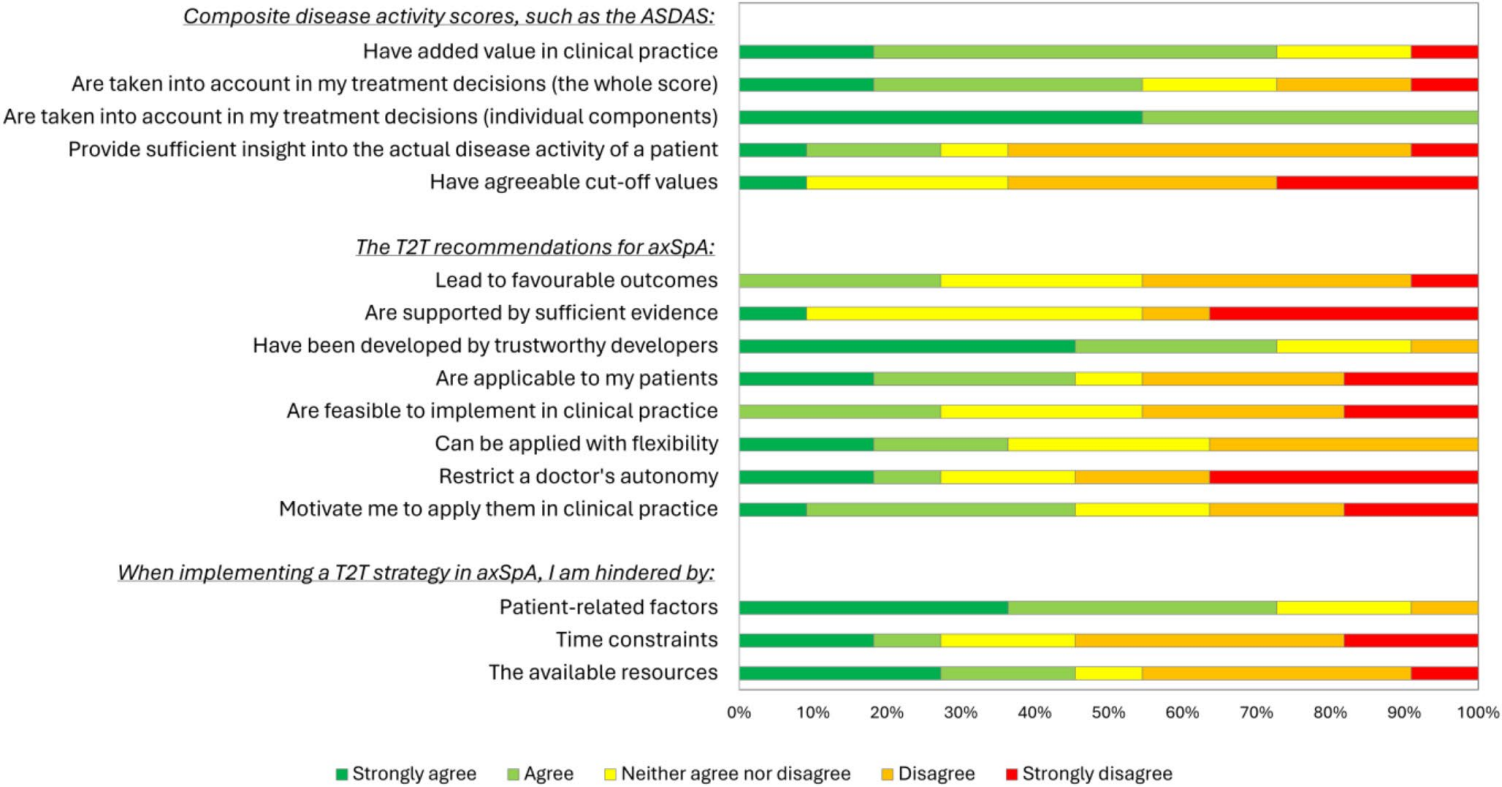
Results of the supplementary survey for rheumatologists (*n* = 11). ASDAS: Axial Spondyloarthritis Disease Activity Score; axSpA: axial spondyloarthritis; T2T: treat-to-target

A non-exhaustive sample of illustrative quotes per identified theme is presented in Table 2 (qualitative component). Survey results from patients and rheumatologists are presented in Figs. 1 and 2, respectively (quantitative component).

**Table 2:** Illustrative quotes from patients and rheumatologists

Themes	Quotes from patients	Quotes from rheumatologists
***Barriers to T2T***		
Disease activity measurement	P1: “When I filled in the questionnaire, I had just gotten the flu vaccine, so I was not feeling so well. And if you then fill it in honestly, you get a completely different score than in a normal situation.”	R1: “[The patient] has absolutely no sacroiliitis, no peripheral arthritis, but still many complaints and therefore a high ASDAS. This has to do with the fact that [the patient] is very active and has mechanical complaints, also due to osteoarthritis.” R2: “If I would apply T2T with everyone according to the ASDAS… I’m not sure. My most important question is, is [the current situation] acceptable for you?”
Patient-related factors	P2: “I always have some pain, but everyone has that. The limitations that I have, I can live with them.” P3: “If nothing changes and I can keep my disease stable, then I have no need for appointments with the doctor.” P4: “My rheumatologist wanted to improve my situation further by increasing my medication or switching to another. […] But I find it difficult to switch, if you read about the side effects that other patients had… I do not look forward to that. […] If you try something new and it does not work, then you have to go back to the old medication anyway.” P5: “It is not that bad, the fluctuations [in disease activity] come every half year in the same way.” P6: “I have had [no medication] in the last years. Why nothing? I go twice a year for six weeks to a warmer country with low humidity. I specially go there to suppress my disease, that does me good.” P7: “In any case, I know that if I have complaints that I can always get in contact. And that works well for me.”	R3: “You always have to think, am I doing harm by not intensifying [the treatment]? Apart from [the patient’s] functioning, is there an increased chance of damage? And the conclusion was that we had no reason to intensify the treatment. […] The advantages of not switching are bigger than the advantages of intensifying treatment.” R4: “I do not explicitly say, this is the treatment goal and this is what we are working towards. Not with this patient, because for [the patient] it would only lead to confusion.” R5: “I am faced with roadblocks due to intolerances and side effects. The idea is always to strive for remission, but sometimes that is just not possible.”
Limited treatment options and evidence		R6: “I do not have many options left. So, I am a bit careful to not just stop the [current] medication like that.” R7: “There are studies available, however, there is no evidence that the choice I made [to not escalate treatment] was wrong. I do not have convincing arguments that I am undertreating my patients [by not applying a T2T strategy] based on the current trials that have been done in axSpA.”
Logistical challenges	P8: “My gastroenterologist is in a different hospital [than my rheumatologist]. As a result, the communication is sometimes more difficult and restricted.”	R8: “In an ideal situation without any time pressure, you can discuss all the pros and cons of different treatment options, but in practice there is no time. I have time for a history taking, sometimes a physical examination and interpretation of the CRP and ASDAS, and to discuss the conclusion with the patient. […] But I do not have time for more.” R9: “Maybe I need to change and move with the times, and use the ASDAS or BASDAI more often. But it simply is not in my routine, and that is hard to change. Support with this would be very helpful, such as in the EMR.”
***Facilitators of T2T***		
Knowledge and awareness	P9: “I know what I have. I know what I can do about it, and what cannot be done.”	
Doctor-patient relationships	P10: “If the doctor says, ‘I would like to see you once a month’, then you naturally go along with that. It is in your own best interest.” P11: “You are the experts. If you go to buy a car, you assume that the salespeople are the experts, but you can still say if you like the colour, as a figure of speech.” P12: “It is always pleasant [to make decisions] together. [My rheumatologist] always asks, ‘do you agree?’, if we are going to do something new. I then understand the reasoning behind it immediately as well.”	R10: “I always try to inform patients and invite them to think along, to find the best option for them. And [to ensure] a clear understanding of what the options are.” R11: “Maybe, as a rheumatologist, I do not know all the possibilities to change behaviour or the lifestyle of older patients that need it. […] [I involved] the informal caregivers, to encourage [the patient] to still walk small distances independently.”
Supporting infrastructure	P13: “I want to know; where do we go from here? How can I help myself further? I got new insights from the nurse in this aspect.”	

ASDAS: Axial Spondyloarthritis Disease Activity Score; axSpA: axial spondyloarthritis; BASDAI: Bath Ankylosing Spondylitis Disease Activity Index; CRP: C-reactive protein; EMR: electronic medical record; T2T: treat-to-target

### Barriers to T2T

Four themes pertaining to barriers were identified. In patients where the rheumatologist found the ASDAS to be falsely elevated and therefore not an indication to intensify treatment, inaccuracies in *disease activity measurement* was a prominent theme. Conversely, in patients where the rheumatologist agreed with the ASDAS indicating active disease and considered treatment adaptations, obstruction by *patient-related factors* or the *limited treatment options and evidence* available to support a T2T strategy in axSpA were highlighted. In addition, *logistical challenges* was a theme applicable to both subgroups.

#### Disease activity measurement

Challenges in accurately measuring disease activity using the ASDAS were emphasised. Particularly, numerous patients reported both during the interviews and in the survey (*n* = 10/16, 62.5%) that their input on disease activity questionnaires was often influenced by comorbidities with overlapping symptoms (Quote P1). This resulted in frequent discrepancies between the ASDAS and the rheumatologist’s clinical impression, instilling doubts on the ability of the ASDAS to discern inflammatory from non-inflammatory symptoms and provide sufficient insight into actual disease activity (*n* = 8/11, 72.7%) (Quote R1). Considering that non-inflammatory comorbidities, such as pain sensitisation syndromes, may present with symptoms that resemble axSpA manifestations (e.g. enthesitis-like symptoms), it was mentioned that using scoring instruments to make this differentiation is particularly challenging in patients with axSpA. To illustrate the magnitude of some of these discrepancies, in certain cases, a de-escalation of therapy was even considered despite an ASDAS indicating active disease due to the absence of inflammatory symptoms on clinical examination. This also relates to concerns expressed by rheumatologists in the survey on the established cut-off values (*n* = 10/11, 90.9%). Therefore, even though rheumatologists acknowledged the value of using (individual components of) the ASDAS for longitudinal monitoring and to aid treatment decisions (*n* = 8/11, 72.7%), especially in the early stages of disease management, achieving a low ASDAS was rarely the sole treatment target pursued for the patients discussed (Quote R2). Rheumatologists preferred to base decisions on their own clinical judgement instead (derived from the patients’ history, physical examination, and laboratory and imaging results), and were reluctant to intensify treatment in the absence of objective manifestations or if a plausible alternative aetiology for the symptoms was present.

An additional consideration was the temporal context of disease activity measurements. Patients reported experiencing frequent fluctuations in symptoms, making single ASDAS measurements not reflective of their overall condition. Furthermore, rheumatologists believed that treatment intensification would have been premature in patients who had recently started new drugs or were awaiting further diagnostics.

#### Patient-related factors

The personal circumstances of patients were frequently central to the decision by rheumatologists to not intensify treatment (*n* = 8/11, 72.7%). A common sentiment amongst patients was satisfaction with the status quo and the wish to continue with their existing treatment regimens (Quotes P2 and P3). As reflected by patients both during the interviews and in the survey, there was also concern about switching drugs, for instance due to previous negative experiences with treatment adaptations, doubts about the effectiveness of other drugs (*n* = 4/16, 25.0%), or fear of side effects of new drugs (*n* = 5/16, 31.3%) (Quote P4). Finally, patients often believed that axSpA flares were temporary and self-limiting (*n* = 12/16, 75.0%), making treatment intensification unwarranted (Quote P5). Overall, the majority (*n* = 13/16, 81.3%) of patients had no desire for treatment adaptations, some of whom expressed a definitive objection. Instead, several stated the preference for analgesics or non-pharmacological interventions to sustain an acceptable symptom state (Quote P6). In such scenarios, rheumatologists were inclined to follow the patient’s lead, provided that the risks of forgoing treatment intensification were acceptable (Quote R3).

Another obstacle was patients’ limited knowledge on the rationale of the T2T strategy and treatment goals. Rheumatologists noted that these topics were rarely discussed explicitly during consultations, and experienced challenges in educating patients on these aspects, particularly in the early stages of disease management when patients might feel overwhelmed (Quote R4). Additionally, patients with underlying learning disabilities or psychiatric comorbidities sometimes had difficulty understanding, or were less receptive to, new treatment-related explanations and instructions. As such, some patients– especially those who considered their symptoms acceptable despite an elevated ASDAS– found tight control unnecessary. Having infrequent follow-up appointments, combined with the possibility to contact the outpatient clinic if needed in-between, was perceived as sufficient by them (Quote P7).

Finally, objective health-related factors, such as drug intolerances, complicated the disease management of some patients and limited the execution of a T2T strategy as well (Quote R5).

#### Limited treatment options and evidence

The limited number of pharmacological treatment options available for axSpA was emphasised by rheumatologists, which led to reluctance to rapidly cycle between different drugs to avoid exhausting all alternatives (Quote R6). As highlighted both during the interviews and in the survey, limited evidence available to support a T2T strategy in axSpA further reinforced this reluctance (*n* = 10/11, 90.9%), as well as scepticism about the benefits of aggressive treatment on patient outcomes (*n* = 8/11, 72.7%). Based on their previous experiences in treating axSpA, rheumatologists did not believe that deviating from T2T recommendations would necessarily lead to undertreatment or poorer outcomes (Quote R7).

#### Logistical challenges

Rheumatologists reflected that time pressure and capacity shortages at the outpatient clinic led to inability to consistently apply all components of T2T, such as the regular evaluation of specific treatment goals (Quote R8). This is consistent with the survey, where rheumatologists expressed doubts on the applicability (*n* = 6/11, 54.5%), feasibility (*n* = 8/11, 72.7%) and flexibility (*n* = 7/11, 63.6%) of a T2T strategy in practice. Additionally, rheumatologists found their current electronic medical records (EMRs) unsuitable for the longitudinal monitoring of disease activity scores and the recording and tracking of treatment goals (Quote R9). Applying a T2T strategy was, therefore, not embedded in the routines of rheumatologists, and the majority expressed low motivation to do so (*n* = 6/11, 54.5%). Finally, one patient who was under treatment by multiple specialists (due to the presence of extra-musculoskeletal manifestations) found that care was not always well-coordinated, leading to difficulties in adapting treatment plans (Quote P8).

### Facilitators of T2T

Three themes pertaining to facilitators were identified, relating to patients’ *knowledge* about axSpA and rheumatologists’ *awareness* of the T2T strategy, positive *doctor-patient relationships*, and availability of a *supporting infrastructure* at the outpatient clinic.

#### Knowledge and awareness

Patients expressed having broad knowledge about axSpA, particularly on manifestations indicating active disease, enabling constructive discussions between patients and their healthcare providers (Quote P9). This insight was facilitated by explanations provided by healthcare providers (often shortly after diagnosis) and the proactive attitude demonstrated by certain patients.

Rheumatologists were largely familiar with T2T recommendations (high awareness: 45.5% [*n* = 5/11], moderate awareness: 54.5% [*n* = 6/11]), and the majority had trust in its developers (*n* = 8/11, 72.7%). Consistent with T2T recommendations, all rheumatologists strived for clinically inactive disease in their patients (albeit not necessarily according to the ASDAS), with attention for quality of life in particular.

#### Doctor-patient relationships

The robust collaboration between patients and rheumatologists was underscored. Patients experienced high involvement of rheumatologists in their care and trusted their expertise. This translated into willingness to follow their rheumatologist’s advice, as long as sufficient justification was provided. Particularly, patients viewed compliance with professional advice as being in their own interest, and were therefore open to tight(er) control if deemed medically necessary (Quote P10).

In addition, positive doctor-patient relationships facilitated constructive SDM. While rheumatologists reflected that not all patients were equipped or motivated to actively participate in SDM, it was still considered a necessary component of consultations (e.g. to provide explanations and discuss patient opinions) (Quote R10). Likewise, patients indicated a widespread preference for SDM over a paternalistic approach, albeit guided by the rheumatologist’s expertise (Quote P11). In practice, both patients and rheumatologists felt that SDM had been well-implemented, which in turn contributed to further strengthening of doctor-patient relationships with time, and reassured patients that their personal concerns were taken seriously (Quote P12).

Finally, one rheumatologist highlighted collaboration with informal caregivers in the SDM process as a pivotal supporting factor, particularly to encourage compliance (Quote R11).

#### Supporting infrastructure

Certain modalities available for disease monitoring were emphasised as beneficial. Patients highly appreciated having continuity of care and the ability to contact the outpatient clinic between consultations. Additionally, some patients recognised the benefits of using disease activity questionnaires as an assisting tool, provided they were addressed during consultations. While it was mentioned during the interviews that scores were frequently biased and therefore required contextualisation with the patient’s history (as highlighted in the theme ‘Disease activity measurement’), patients expressed in the survey that these instruments were, to a certain extent, still reflective of their current condition (*n* = 12/16, 75.0%). Finally, the availability of digital resources and possibility for consultations with rheumatology nurses to offer further guidance on the use of anti-inflammatory drugs, non-pharmacological therapies, and lifestyle was valued (Quote P13).

## Discussion

In this study, challenges in using the ASDAS, and numerous patient-related factors such as concern about treatment adaptations, were identified as key barriers to T2T in axSpA. The limited number of viable treatment options and scarce amount of evidence supporting a T2T strategy in axSpA, as well as logistical challenges, were additional obstacles. Conversely, facilitators included patients’ broad knowledge about axSpA, rheumatologists’ awareness of T2T recommendations, positive doctor-patient relationships, and the presence of a supporting infrastructure.

Taking into account the substantial barriers identified, it is worth reflecting on the practicality and desirability of a T2T strategy in axSpA– a dilemma already highlighted by previous studies and certain treatment guidelines [13, 20–22]. In particular, the concern held by patients about treatment adjustments when symptoms were considered acceptable (despite incomplete suppression of disease activity) is a barrier that we observed which aligns with past studies on T2T implementation in rheumatology practice [23–25]. Additionally, the barriers posed by interfering biological or psychosocial factors noted in this study, such as drug intolerances, fear of side effects of new drugs and low receptiveness to new instructions, have been described before [23–25]. The limited organisational capacity to execute a T2T strategy in rheumatology practice must also not be overlooked [26–28]. These challenges are further compounded by the lack of definitive evidence demonstrating that a T2T strategy is beneficial in axSpA, especially when combined with the suggestion from previous studies that aggressive treatment is not always necessary to maximise patient health. For example, the association between radiographic progression and functional impairment is unclear, inferring that a T2T strategy might not necessarily result in better outcomes [29, 30]. Given these fundamental concerns, it is uncertain whether the current form of the T2T strategy is optimal in axSpA, or if a modified approach, such as a dual-target strategy where symptom-guided treatment is emphasised alongside attaining biological remission, should take preference [22, 31]. Irrespective of which strategy future guidelines endorse, it is crucial for successful implementation that all stakeholders agree on the chosen approach [14].

Another evident dilemma in the management of axSpA is determining how treatment targets should be measured. While it is logical that disease activity should be considered (with ID/LDA as the preferred target), rheumatologists acknowledged that achieving this goal as currently defined (i.e. ASDAS < 2.1) is not always feasible [12]. This is consistent with the outcomes of randomised controlled trials on bDMARDs in axSpA, which have highlighted that achieving an ASDAS < 2.1 might be unrealistic for many patients [32–34]. Difficulty in achieving a low ASDAS may, for example, be attributed to the high occurrence of symptoms such as non-inflammatory pain and stiffness even after inflammation has subsided, or to activity of overlapping comorbidities [35, 36]. It is known that patients struggle to discern inflammatory from non-inflammatory complaints, leading to inconsistent ASDAS outputs, as we also observed [37]. Consequently, in the absence of objective symptoms, or if the symptom state is acceptable in the patient’s perspective, rheumatologists remain cautious to base decisions solely on the ASDAS and similar composite scores, as this approach may oversimplify the multifaceted and heterogeneous nature of axSpA and lead to the risk of overtreatment [21, 24, 38, 39]. These limitations in the ASDAS suggest the need to re-evaluate the definition and assessment of active disease in axSpA in the context of T2T.

Reassuringly, several factors that could enable the successful implementation of a (form of the) T2T strategy in axSpA are already in place. Similar to previous findings in RA, patients’ broad knowledge about axSpA and positive doctor-patient relationships are particularly beneficial [40]. Interestingly, while past studies identified low familiarity with T2T recommendations amongst rheumatologists as a barrier, our results contradict this, as awareness was generally high in our cohort [26, 41]. While this awareness may be attributed to increasing attention on T2T strategies in recent years, and the fact that this study was conducted at SpA and T2T expertise centres, this is nonetheless an encouraging finding. Overall, if a T2T strategy is to be endorsed as the way forward, these are examples of facilitators that should be leveraged.

The primary strength of this study is that it is, to our knowledge, the first specifically designed to explore the barriers and facilitators to a T2T strategy in axSpA in clinical practice, leading to relevant and applicable findings. Furthermore, our mixed methods design included both patients and rheumatologists, allowing for diverse perspectives to be captured. Finally, the clinical setting of this study, combined with its multicentre design with comparable findings in both participating hospitals, make our results generalisable to Dutch rheumatology practice. However, several limitations should also be acknowledged. Firstly, the time between the consultation and interview was delayed by a few weeks for some participants, which may have introduced recall bias. Secondly, the mean symptom duration of the patients who participated was considerably long. It is therefore plausible that the prevalence of non-inflammatory symptoms from structural damage or degenerative disorders in our cohort was relatively high, possibly leading to an overemphasis on certain barriers [42]. Lastly, as only stakeholders from the Netherlands were included, our findings may not be equally applicable to other countries.

Despite these limitations, our results provide deeper insights into the applicability of a T2T strategy in axSpA from a real-world perspective. Our findings may also inform future research on several aspects. Firstly, it needs to be determined if a T2T strategy is effective in axSpA through additional clinical trials. Secondly, EMRs that are better suited to facilitate a T2T strategy need to be developed (e.g. with added functionality such as automated collection of patient-reported outcomes and tracking of treatment goals through user-friendly, personalised mobile apps integrated within hospital EMRs) [43, 44]. Thirdly, future studies should explore how patient education regarding the importance of adequate disease management can be enhanced, and which methods can be used to more effectively engage patients in the application of T2T strategies (e.g. in the determination of treatment goals and follow-up frequency). Ideally, such guidance strategies should also account for contextual factors which may influence the disease management process of individual patients, including factors that were not investigated in the present study such as level of educational attainment. Improvements in this regard could lead to better compliance and satisfaction with care amongst patients [45]. Finally, the subgroups of patients who face obstacles in the application of a T2T strategy, such as those with factors that interfere with disease activity assessment or patients with ‘difficult-to-manage’ disease, should be investigated further to enable individualised and effective disease management [46].

In conclusion, numerous barriers and facilitators to the implementation of a T2T strategy in axSpA are present. These factors need to be considered in the future when applying T2T strategies in practice.

## Electronic supplementary material

Below is the link to the electronic supplementary material.

Supplementary Material 1

Supplementary Material 2

Supplementary Material 3

Supplementary Material 4

## Data Availability

The data that support the findings of this study are available from the corresponding author upon reasonable request.

## Declarations

### Disclaimer

No part of this manuscript, including text and graphics, are copied or published elsewhere in whole or in part.

### Note on related congress abstracts and publication

This work has previously been presented as poster presentations at the EULAR 2024 Congress (Vienna, Austria, 2024), 14^th^ International Congress on Spondyloarthritides (Ghent, Belgium, 2024) and Dutch Society for Rheumatology Najaarsdagen 2024 (Arnhem, The Netherlands, 2024). Abstracts have been published in Annals of the Rheumatic Diseases (10.1136/annrheumdis-2024-eular.565, abstract number: POS0802) and Clinical and Experimental Rheumatology (10.55563/clinexprheumatol/7l5roe, abstract number: P101).
