# Supplementary material for: Barriers and facilitators to treat-to-target in axial spondyloarthritis in clinical practice: a mixed methods study
Source: Rheumatol Int. 2025 Jan 31;45(2):41. doi: 10.1007/s00296-025-05795-6 (PMC11785688; doi:10.1007/s00296-025-05795-6)
Supplement: Supplementary file 1 — Supplementary Material 1 [file 296_2025_5795_MOESM1_ESM.pdf]

Online Resource 1: Interview guide for patients

Questions regarding disease characteristics:

1. How long ago was your disease diagnosed? If <5 years ago:
    a. Did you receive an explanation about the disease axial spondyloarthritis from your doctor and/or nurse? And about the treatment? And what you could do yourself to manage the disease? How soon after you received the diagnosis did this take place?
    b. Did you receive information about the advantages of regular physical activity and exercise?
2. How often do you visit the outpatient clinic for your rheumatic disease? What do you think of this frequency?
3. What is your understanding of the concept, 'disease activity'?

Questions regarding disease management:

4. Did you receive information about the goal of a pharmacological or non-pharmacological treatment? If yes, what information did you receive? Who did you receive this from? Which treatment goal(s) were discussed with you?
5. Did you receive information about the importance of a pharmacological or non-pharmacological treatment? If yes, what information did you receive? Who did you receive this from?
6. Has a manner of measuring disease activity been agreed with you? Such as filling in questionnaires or doing blood tests?
7. If you filled in questionnaires, what was your opinion on these questionnaires? Were your answers discussed with you?
8. Patients who have high disease activity are advised to have a follow-up visit at the outpatient clinic within 3 months. Was this the case for you?
9. It is sometimes advised that patients with high disease activity visit the outpatient clinic every 4 weeks. What do you think of this?
10. Did you receive information about the advantages of a pharmacological treatment? And about the risks or possible side effects?
11. Do you think that your disease can be brought under control? Why or why not?
12. How aware are you of your ability to participate in the decision-making process during your visits to the outpatient clinic?
13. How do you view your involvement in the decision-making process?
14. Do you prefer making decisions together with your doctor, or would you rather have your doctor make the decisions?
15. What (more) would you need to be able to contribute to decision making with your doctor?
16. Even though you currently have high disease activity, no changes were made to your pharmacological treatment. Why is this?
