# Supplementary material for: Barriers and facilitators to treat-to-target in axial spondyloarthritis in clinical practice: a mixed methods study
Source: Rheumatol Int. 2025 Jan 31;45(2):41. doi: 10.1007/s00296-025-05795-6 (PMC11785688; doi:10.1007/s00296-025-05795-6)
Supplement: Supplementary file 2 — Supplementary Material 2 [file 296_2025_5795_MOESM2_ESM.pdf]

Online Resource 2: Interview guide for rheumatologists

Questions regarding each patient case (repeated per patient):

1. What is your treatment goal for this patient?
2. If applicable, do you also take extra-musculoskeletal manifestations into consideration in the treatment goal for this patient?
3. Did you decide on this treatment goal together with the patient?
4. How realistic do you think achieving this treatment goal is for this patient?
5. How do you determine if the treatment goal is achieved? Do you use a measurement tool for this? If yes, which one?
6. Do you measure inflammation through blood tests? If yes, do you do this at every visit?
7. Which measurement is decisive for you in determining when the treatment goal has been achieved?
8. How often do you evaluate the treatment goal? What do you base your evaluation on?
9. Even though the ASDAS is elevated for this patient, do you think that this patient is in remission? Or has low disease activity?
10. When did you last change the treatment for this patient?
11. If the patient uses a biological DMARD, which factors did you consider in the decision to start with a biological?
12. Even though there is high disease activity according to the ASDAS, no changes to the patient's pharmacological treatment took place. Has the possibility to change treatment been discussed with the patient? Why was the pharmacological treatment not changed?

Questions regarding shared decision-making:

13. What are your thoughts on shared decision-making?
14. What do you think your role is in shared decision-making?
