# Supplementary material for: Barriers and facilitators to treat-to-target in axial spondyloarthritis in clinical practice: a mixed methods study
Source: Rheumatol Int. 2025 Jan 31;45(2):41. doi: 10.1007/s00296-025-05795-6 (PMC11785688; doi:10.1007/s00296-025-05795-6)
Supplement: Supplementary file 3 — Supplementary Material 3 [file 296_2025_5795_MOESM3_ESM.pdf]

Online Resource 3: Survey for patients

**Age**	
**Sex**	
**Year in which symptoms began**	

**Question**	**Cross one**		
	**Not at all**	**Maybe**	**Very much**
How much would you like your treatment to be changed?			

**Statement**	**Cross one**		
	**Disagree**	**Agree**	**Not applicable**
It is clear to me that my disease activity is high based on the questionnaire.			
The outcomes of the questionnaire about my disease activity were discussed with me during consultations.			
The doctor does not want to change my treatment (yet).			
I do not want my treatment to be changed (yet).			
I expect that my high disease activity is temporary.			
I recently started with a new drug, and my doctor and I are still anticipating further effects from it.			
The outcomes of the questionnaire about my disease activity do not reflect how active my disease actually is. In reality, my disease activity is lower.			
The outcome of the questionnaire is influenced by other factors unrelated to my rheumatic disease.			
I do not have trust in new drugs.			
I am afraid to start new drugs.			
I do not expect that a change in my drugs will be effective in improving my symptoms.			
I do not take my prescribed drugs consistently.			
I cannot start a new treatment due to other diseases that I have.			
There are no alternative treatment options available anymore for my disease.			
