# Supplementary material for: Barriers and facilitators to treat-to-target in axial spondyloarthritis in clinical practice: a mixed methods study
Source: Rheumatol Int. 2025 Jan 31;45(2):41. doi: 10.1007/s00296-025-05795-6 (PMC11785688; doi:10.1007/s00296-025-05795-6)
Supplement: Supplementary file 4 — Supplementary Material 4 [file 296_2025_5795_MOESM4_ESM.pdf]

Online Resource 4: Survey for rheumatologists

**Age**	
**Sex**	
**Years as a practicing rheumatologist**	

**Question**	**Cross one**		
	**Not at all**	**Somewhat**	**Very**
How aware are you of the treat-to-target (T2T) recommendations for axSpA? (For example, from articles or congresses?)			

Questions regarding composite disease activity scores (e.g. ASDAS):

**Statement**	**Cross one**				
	**Strongly disagree**	**Disagree**	**Neither agree nor disagree**	**Agree**	**Strongly agree**
I see an added value of these measurement instruments in clinical practice.					
I take the results of these measurement instruments (as a whole) into account in my treatment decisions.					
I take individual components of these measurement instruments into account when deciding on a treatment plan.					
These instruments provide me with insufficient insight into the actual disease activity of a patient.					
I agree with the cut-off values used by these instruments to determine low or high disease activity.					

Questions regarding T2T in axSpA:

**Statement**	**Cross one**				
	**Strongly disagree**	**Disagree**	**Neither agree nor disagree**	**Agree**	**Strongly agree**
There is sufficient evidence regarding T2T in clinical practice.					
The T2T recommendations are applicable to my patients.					
I have trust in the developers of the T2T recommendations.					
The T2T recommendations can be applied with flexibility.					
The T2T recommendations do not restrict a doctor’s autonomy.					

The T2T recommendations lead to favourable outcomes.					
The T2T recommendations can be feasibly implemented in clinical practice.					
I am motivated to apply the T2T recommendations in clinical practice.					
I am hindered in applying T2T in clinical practice due to patient-related factors.					
I do not have time to apply the T2T recommendations in clinical practice.					
I do not have enough resources available to apply the T2T recommendations.					
